# CARP-1 / CCAR1: A biphasic regulator of cancer cell growth and apoptosis

**DOI:** 10.18632/oncotarget.3376

**Published:** 2015-03-24

**Authors:** Magesh Muthu, Vino T. Cheriyan, Arun K. Rishi

**Affiliations:** ^1^ John D. Dingell VA Medical Center, Wayne State University, Detroit, MI, USA; ^2^ Karmanos Cancer Institute, Wayne State University, Detroit, MI, USA; ^3^ Department of Oncology, Wayne State University, Detroit, MI, USA

**Keywords:** CARP-1/CCAR1, CFMs, apoptosis

## Abstract

Targeted cancer therapy using small molecule inhibitors (SMIs) has been useful in targeting the tumor cells while sparing the normal cells. Despite clinical success of many targeted therapies, their off-target effects and development of resistance are emerging as significant and challenging problems. Thus, there is an urgent need to identify targets to devise new means to treat cancers and their drug-resistant phenotypes. CARP-1/CCAR1 (Cell division cycle and apoptosis regulator 1), a peri-nuclear phospho-protein, plays a dynamic role in regulating cell growth and apoptosis by serving as a co-activator of steroid/thyroid nuclear receptors, β-catenin, Anaphase Promoting Complex/Cyclosome (APC/C) E3 ligase, and tumor suppressor p53. CARP-1/CCAR1 also regulates chemotherapy-dependent apoptosis. CARP-1/CCAR1 functional mimetics (CFMs) are a novel SMIs of CARP-1/CCAR1 interaction with APC/C. CFMs promote apoptosis in a manner independent of p53. CFMs are potent inhibitors of a variety of cancer cells including the drug (Adriamycin or Tamoxifen)-resistant breast cancer cells but not the immortalized breast epithelial cells, while a nano-lipid formulation of the lead compound CFM-4 improves its bioavailability and efficacy *in vivo* when administered orally. This review focuses on the background and pleiotropic roles of CARP-1/CCAR1 as well as its apoptosis signaling mechanisms in response to chemotherapy in cancer cells.

## INTRODUCTION

According to “World Cancer Report 2014” by WHO, in 2012 about 14.1 million new cases of cancer occurred globally and caused 14.6% of all human deaths [1]. It is expected that annual cancer cases will rise from 14.1 to 22 million within the next two decades [1]. Disturbingly, cancer remains the second most common cause of death among children between the ages of 1 to 14 years in the US [1]. Over the past years, with the help of well adapted genomic and proteomic technologies, researchers have made strides in better understanding the biochemical and molecular mechanisms of tumorigenesis. Conventional radio and chemo therapies, coupled with newer targeted cancer therapy are currently utilized for management and treatment of a variety of cancers in the clinic. Although a full understanding of the molecular complexity of cancers is yet to be realized, considerable progress have been made in the treatment of a range of cancers that include chronic myeloid leukemia (CML), acute promyelocytic leukemia (APL), Hodgkin's lymphoma and testicular cancers [2]. Unfortunately, the successful outcome of many anti-cancer treatments and disease-free survival of the patients is often short lived due in part to limiting toxic side effects of therapies as well as emergence of drug-resistant cancers. Therefore, it is necessary to discover new targets and molecular pathways that regulate processes of carcinogenesis in order to improve diagnoses and therapeutic efficacy of current drugs as well as to permit design and development of novel therapeutic strategies to effectively combat cancers including their drug-resistant variants.

### Discovery of CARP-1/CCAR1

By utilizing a functional gene knockout technology, CARP-1/CCAR1 was originally identified as a peri-nuclear phospho-protein that was required for apoptosis signaling by a retinoid (CD437) as well as chemotherapeutics Adriamycin (ADR) and Etoposide in the Human Breast Cancer (HBC) cells [3]. The current GenBank database indicates nucleotide sequences encoding CARP-1/CCAR1 proteins of various species including Mus musculus (mouse), Canis lupus (dog), Rattus norvegicus (rat), Pan troglodytes (chimpanzee), Gallus gallus (domestic chicken), Danio rerio (zebrafish), Xenopus laevis, Caenorhabditis elegans (worm) and Ornithorhynchus anatinus (platypus). Human CARP-1/CCAR1 gene is located at the long arm of chromosome 10 (10q21–10q22), and encodes a transcript of ~3.5 kilobase (kb) size. The CARP-1/CCAR1 protein has 1150 amino acid residues with approximate molecular mass of 130 kiloDaltons (kDa). A number of studies to date have revealed CARP-1/CCAR1 involvement in cell proliferation as well as apoptosis signaling in a variety of cell types including different cancer cells [4, 5].

### CARP-1/CCAR1 as a regulator of steroid/thyroid receptor signaling

Nuclear receptors (NRs) are highly conserved family of transcription factors that regulate transcription of target genes in ligand–dependent manner. NRs include receptors for estrogen (ER, NR3A), progesterone (PR, NR3C3), vitamin A (RAR, NR1B), vitamin D (VDR, NR1I1), androgen (AR, NR3C4), and thyroid hormones (TR, NR1A). NRs bind to specific DNA sequences in response to their ligand binding, which in turn recruits other co-activators that remodels the chromatin in regulating RNA polymerase II associated basal transcriptional mechanism [6]. For example, P160 family of coactivators that include Steroid receptor coactivator 1 (SRC-1; also known as nuclear receptor coactivator-1; NCoA-1), SRC-2 (also known as Glutamate receptor interacting protein 1; GRIP1, Transcriptional intermediary factor 2; TIF2 or NcoA-2), and SRC-3 (also known as ACTR, Amplified in breast 1; AIB1, pCIP, RAC3, or Thyroid hormone receptor activator molecule 1; TRAM1) harbor multiple leucine rich repeat (LXXLL) motifs, which promote ligand-dependent interactions with the activation function 2 (AF2) domain of the NRs [6]. The p160 coactivators also recruit multiple acteyltransferases including CBP/p300 and P300-CBP associated protein (PCAF) and methyltransferase coactivators such as coactivator-associated arginine methyl transferase 1 (CARM1) and Protein arginine methyl transferase 1 and 2 (PRMT1 & 2) as well as other downstream targets including coiled-coil coactivator (CoCoA) [7, 8]. Some nuclear hormone receptors such as ERα regulate activator protein (AP)-1 dependent gene expression by directly binding with c-Jun and JunB proteins but not with c-fos proteins [9].

In 2008, Kim et al. reported that CARP-1/CCAR1 functions as co-activator of NRs. CARP-1/CCAR1 associates with components of Mediator and p160 co-activator complexes and is recruited to endogenous NR target genes in the presence of appropriate hormone. CARP-1/CCAR1 interacts with and co-operates with CoCoA and GRIP1 co-activators for estrogen receptor (ER) signaling, and promotes growth of MCF-7 HBC cells in response to estradiol (E2) treatment [10]. CARP-1/CCAR1 also physically and functionally associated with mediator complex including NUT2/MED10 and MED23 [11]. CCAR1/CARP-1 stimulated the basal and hormone dependent activities of glucocorticoid (GR) and thyroid hormone receptor β1 (TR) reporter genes in CV-1 cells [10]. Besides co-activation of GR, CARP-1/CCAR1 also positively regulated adipogenesis through GR signaling pathway [11]. A recent investigation also revealed that carboxyl terminal region of the transcription factor GATA1 interacts with CARP-1/CCAR1, CoCoA, and MED1. CARP-1/CCAR1 and CoCoA in fact synergistically enhanced GATA1-mediated transcription from the γ-globin promoter during erythroid differentiation [12].

Additionally, CARP-1/CCAR1 binds directly with ER and associates with p160 coactivators and in turn recruits mediator MED1/TRAP220 (Thyroid hormone receptor associated Protein 220) to ER target gene pS2 in E2-dependent manner. A recent investigation emphasized that CARP-1/CCAR1 is required for growth of prostate cancer cells in part by functioning as a coactivator of AR transcription. CARP-1/CCAR1 plays a crucial role in recruiting GATA2, a key mediator of transcription by AR, in androgen dependent signaling [13]. Knockdown of endogenous CARP-1/CCAR1, compromised the hormone dependent recruitment of mediator complexes to NRs, and consequently impacted growth of hormone dependent cancer cells [10]. Another recent study revealed CARP-1/CCAR1 is a binding partner of β-catenin and enhances the transcriptional activation of β-catenin-Wnt target genes, and mediates anchorage independent growth of colon cancer cells. Depletion of CARP-1/CCAR1 or β-catenin attenuated ability of the colon cancer cells to form colonies in soft agar [14].

Thus, significant investigations suggest that CARP-1/CCAR1 plays important roles in regulating cancer cell growth in part by recruiting multiple mediators and confers optimal conformation for transcriptional functions of NRs. Additionally CARP-1/CCAR1 co-activates the pro-apoptotic tumor suppressor p53 in chemotherapy (ADR)-treated cells. Co-activation of p53 and NRs by CARP-1/CCAR1 highlights its broader biphasic roles in regulating cell growth and apoptosis signaling [10] that are discussed below.

### DBC1, a paralog of CARP-1/CCAR1

Deleted in breast cancer 1 (DBC1), also known as CCAR2, is a nuclear protein encoded by the KIAA1967 gene and is considered as an important paralog of CARP-1/CCAR1 gene. DBC1 was originally discovered and cloned from short arm of chromosome 8 (8p21), which was homozygously deleted in a subset of breast cancers [15]. DBC1 interacts with and negatively regulates SIRT1 deacetylase activity [16, 17]. Subsequent studies revealed that SIRT1 inhibition by DBC1 results in increased acetylation of p53 that, in turn, mediates p53-dependent apoptosis following DNA damage [16, 17]. Inhibition of SIRT1 by DBC1in the DNA damage response (DDR) depends on ATM-dependent Chk2 activation [18]. This Chk2 activation in turn promotes phosphorylation of the 11S proteasome activator REGγ, which increases REGγ–DBC1 interaction and SIRT1 inhibition [18]. Caspase-dependent processing of DBC1 has also been implicated in its pro-apoptotic activity during tumor necrosis factor alpha (TNFα)-mediated apoptosis [19]. However DBC1 loss also promotes death of breast cancer cells [20] while its expression is associated with distant metastatic relapse in patients after receiving endocrine therapy, and thus is an indicator of poor prognosis in a subset of patients [21, 22]. A broader function of DBC1 in metabolism, aging and cancer has recently been highlighted in an elegant review by Chini et al. [23]. Overall, studies thus far suggest that like CARP-1/CCAR1, DBC1 could also play biphasic roles in proliferation and apoptosis signaling.

Although CARP-1/CCAR1 and DBC1 proteins are paralogs, a recent report revealed that both the DBC1 and CARP-1/CCAR1 are largely disordered proteins that evolved from a single common ancestor, the nematode Caenorhabditis elegans protein lateral signaling target (LST)-3 [24]. DBC1 emerged later in evolution than CARP-1/CCAR1 and first appeared in Danio rerio (zebrafish). Interestingly, the DBC1 gene is not present in insects, amphibians, or birds [24]. Nevertheless, a synergistic co-regulation of CARP-1/CCAR1 by DBC1 in mediating ERα target gene transcription in MCF7 and T47D HBC cells was noted in a recent study [25]. DBC1 and CARP-1/CCAR1 cooperatively enhanced and co-activated the ligand-dependent transcription functions of multiple NRs including AR, GR and TR. Depletion of either DBC1 or CARP-1/CCAR1 or both attenuated transcription of ERα target genes [25]. With reference to the co-activator functions, a genome-wide comparative analysis following knock-down of CARP-1/CCAR1 or DBC1 revealed that each co-regulator regulates only a select subset of physiological pathways controlled by glucocorticoids, and the gene-specific actions of these co-activators correspond to specific physiological pathways [26]

### Role of CARP-1/CCAR1 in physiological development

Besides regulating cancer cell growth by co-activating ligand-dependent transcriptional functions of NRs, a number of reports indicate that CARP-1/CCAR1 also co-activates signaling by transcription factors for the normal physiological development. Noggin (Ngn)3 is a helix-loop-helix transcription factor that plays a key role in mediating endocrine differentiation. Notably, Ngn3 regulates pancreatic cell proliferation and differentiation processes and thus serves as a key determinant in pancreatic cell fate during development. CARP-1/CCAR1 binds with Ngn3 in nucleus and co-activates its downstream target gene NeuroD to regulate endocrine differentiation in pancreas during development [27]. Therefore, a broader set of transcriptional co-activation roles of CARP-1/CCAR1 in cell growth, metastasis, and differentiation processes can be deduced on the basis of its ability to co-activate NRs, β-catenin, and Ngn3 [8, 15, 27].

Another recent study revealed modulation of CARP-1/CCAR1 expression and distribution by parathyroid hormone (PTH) in osteoblast differentiation [28]. PTH regulates both anabolic and catabolic process in osteoblast growth and differentiation, and regulates translocation of CARP-1/CCAR1 from nucleus to cytoplasm in remodeling and development of bone tissue. It is well established that Wnt-β-catenin signaling is required for bone physiology [29–31]. Moreover, PTH induces β-catenin activation in multiple cells including UMR, MC3T3-E1 and SAOS [32–35]. Considering the ability of CARP-1/CCAR1 to co-activate Wnt-β-catenin target gene transcription, it is conceivable that CARP-1/CCAR1 could serve as a docking protein to finely regulate β-catenin signaling and apoptosis in the presence of PTH in remodeling bone tissue and development.

Necdin, a member of the MAGE (Melanoma Antigen) protein family, binds with CARP-1/CCAR1, and promotes proteosomal degradation of pro-apoptotic CARP-1/CCAR1 in turn enhancing myoblast differentiation and survival [36]. Interestingly, Necdin also functions as a transcriptional repressor of p53 and inhibits apoptosis [37] regulated by TNFα signaling in preventing muscle atrophy [38]. In light of the fact that CARP-1/CCAR1 co-activates pro-apoptotic transcriptional function of p53, it appears that by negatively regulating CARP-1/CCAR1 Necdin counter-acts the pro-apoptotic activities of p53 and thus contributes to myoblast survival. Additionally, CARP-1/CCAR1 serves as a binding partner of AKAP350A, a multifunctional scaffolding protein, present in Golgi apparatus and centrosomes, along with RAS-GAP SH3 domain binding protein (G3BP), a stress granule marker, and Caprin-1 (Cytoplasmic activation/proliferation-associated protein 1). The oxidative stress following arsenite treatment results in translocation of CARP-1/CCAR1-AKAP350A-G3BP-Caprin-1 complex to RNA stress granules [39]. Since G3BP is known to protect cellular mRNA under stress conditions [40], and CARP-1/CCAR1 protein harbors a cold-shock RNA-binding domain [3], it appears that CARP-1/CCAR1 association with AKAP350A/G3BP/Caprin-1 complex in stress granules likely functions to sequester RNAs to maintain stability and integrity of cellular RNAs in a microtubule-dependent manner during the conditions of cellular stress [39].

### CARP-1/CCAR1 as a regulator of stress-dependent apoptosis

Apoptosis, a process of programmed cell death, plays a pivotal role in maintaining proper tissue homeostasis. Deregulation of apoptosis contributes to various pathological conditions including inflammation and tumorigenesis. Apoptosis is considered as an important attribute of the chemotherapy drugs and serves to eliminate the damaged cancer cells [41, 42]. However, resistance to therapy-induced apoptosis is often encountered in the clinic and contributes to failure in therapy response in cancer patients and results in poor prognosis. Therefore, urgent efforts are warranted to identify additional perhaps new druggable targets to improve therapy efficacy and overcome the resistance mechanisms.

**Figure 1:**
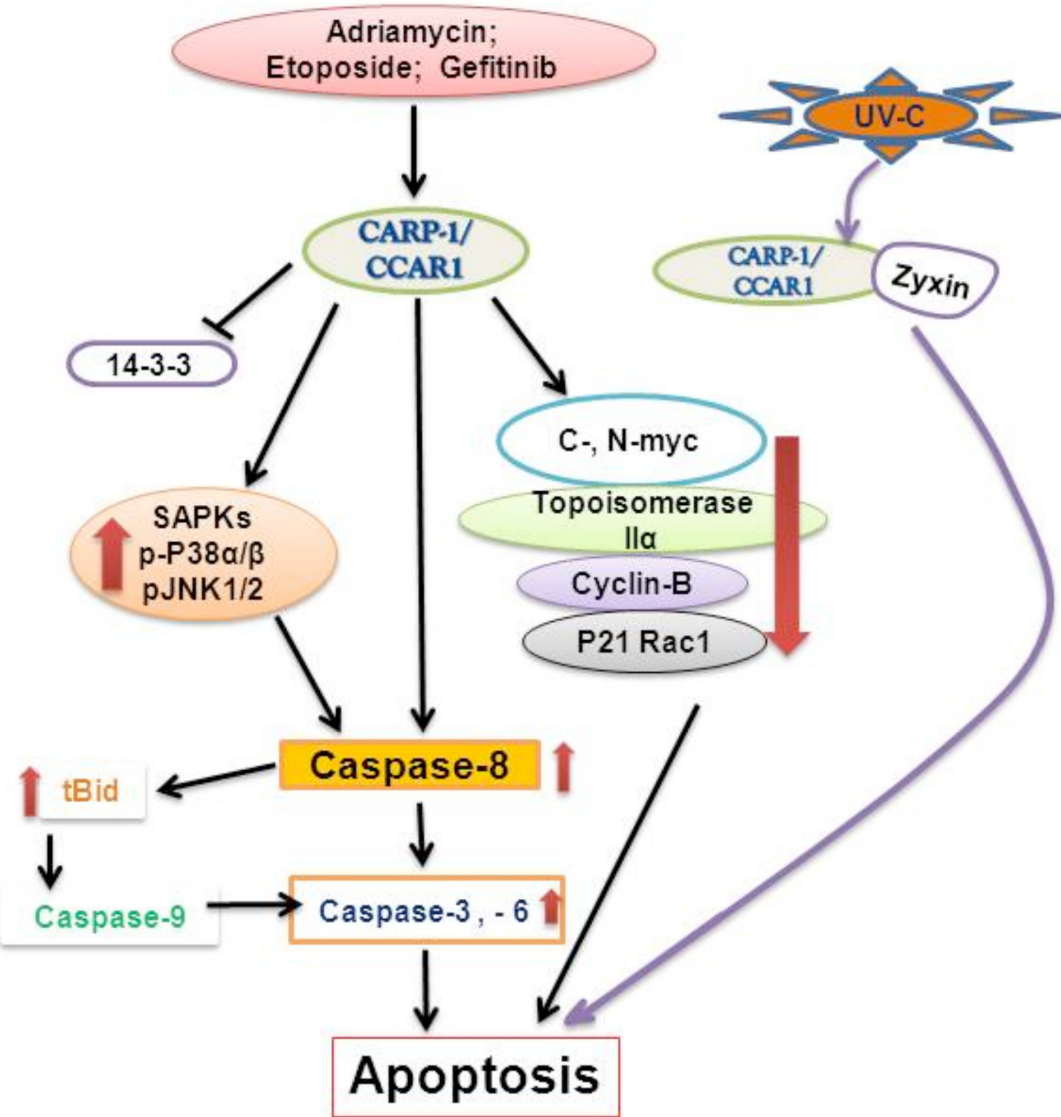
A Schematic of CARP-1/CCAR1 Apoptosis Signaling

As mentioned earlier, CARP-1/CCAR1 was originally indentified and characterized as perinuclear phosphoprotein that was required for apoptosis signaling by chemotherapy drugs ADR and Etoposide independent of the p53 status of the HBC cells [3]. As summarized in figure 1, apoptosis induction by CARP-1/CCAR1 involves its binding with 14–3-3 protein, stimulation of CDKI p21WAF1CIP1 levels and down-regulation of cell growth and cell cycle regulators c-Myc, topoisomerase IIα, cyclin B and p21 Rac1 [3]. Genetic studies revealed that C. elegans Lst3, an ortholog of human CARP-1/CCAR1, is a transducer of Notch signaling. Lst3 also functions as an inhibitor of the EGFR-MAPK pathway [43]. These findings support our earlier studies that demonstrated CARP-1/CCAR1 requirement for apoptosis signaling following EGFR blockage in HBC cells [4]. Apoptosis signaling following EGFR inhibition invloves CARP-1/CCAR1 phosphorylation at tyrosine (Y)^192^, activation of the stress-activated protein kinase (SAPK) p38α/β and caspase 9 [4]. Interestingly, phosphorylation of Y^189^ of murine CARP-1/CCAR1 that corresponds with the Y^192^ of the human homolog has been noted in a global screening of p-tyrosine profile of Src-transformed MEFs [44]. It is likely that a context-dependent tyrosine phosphorylation of CARP-1/CCAR1 plays an important role in signaling for tumor growth or apoptosis, and a thorough functional characterization is needed for further understanding of the mechanism(s) involved. Pharmcologic inhibition of Protein kinase A (PKA) resulted in suppression of HBC cell growth in part by targeting CARP-1/CCAR1 threonine (T)^667^-dependent reduced c-Myc transcription [5]. A number of previous studies have revealed serine (S), and/or threonine (T) as well as tyrosine (Y) phosphorylation of CARP-1/CCAR1 by a variety of different signaling pathways [45–47]. CARP-1/CCAR1 was also found to be a nuclear protein in HeLa cells where it was phosphorylated at its Carboxyl terminus S^1149^, although the precise signaling context was not elucidated [45]. A compilation of the CARP-1/CCAR1 residues modified by phosphorylation and ubiquitination culminating from various proteomic and signaling studies thus far is available at http://www.phosphosite.org. Of note is that signaling by RIP3 kinase, oncogenic phosphatase CDC25B, Aurora and Polo-like kinases phosphorylate CARP-1/CCAR1 T^627^ [48–51], however, no kinase has been identified that directly phosphorylates CARP-1/CCAR1 Y^192^, T^627^, T^667^, or S^1149^.

Epigenetic modifications such as CpG island methylation, histone modification, and deregulation of DNA binding proteins are as important as gene mutation in the process of tumorigenesis. Such epigenetic alterations could either silence key tumor suppressor(s) or promote activation of oncogene(s). In this context our prior reports revealed that CARP-1/CCAR1 expression correlated inversely with the grades of human tumors of breast and lymphoma origins [52, 53]. Although a limited number of specimens were analyzed, diminished expression of CARP-1/CCAR1 in human breast cancers involved methylation-dependent gene silencing that was associated with hypermethylation of CARP-1/CCAR1 promoter [52]. On the other hand, HBC cells stably overexpressing CARP-1/CCAR1 formed reduced sized subcutaneous tumors compared with their wild-type counterparts when xenografted in the immunocompromised SCID mice [52].

A recent study reported that Par-4 (prostate apoptosis response factor-4) and THAP1, a sequence specific DNA binding factor, synergestically cooperate to enhance CARP-1/CCAR1 expression, and promote T-cell acute lymphoblastic leukemia cells (T-ALL) apoptosis [54]. It is known that deregulation of Notch signaling contributes to generation of T-ALL [55]. Here, Par-4/THAP1 complex and Notch 3 competitively regulate alternative splicing of CARP-1/CCAR1 mRNA and impact the T-ALL cell survival. Thus exogenous expression of Notch-ICD (intracellular domain) results in production of truncated form of CARP-1/CCAR1 protein of ~90 kDa along with the full length CARP-1/CCAR1 protein of ~130 kDa, and inhibits Par-4/THAP1 induced CARP-1/CCAR1 expression and apoptosis. Silencing of Notch resulted in the disappearance of truncated ~90 kDa form of CARP-1/CCAR1 and restoration of apoptosis [54]. Although a truncated variant of CARP-1/CCAR1 appears to function as an inhibitor of apoptosis signaling by wild-type CARP-1/CCAR1 in T-ALL cells, ectopic expression of retroviral TAT-domain fusions of a number of non-overlapping CARP-1/CCAR1 peptides that accumulated in the cytosolic compartment, inhibited growth of HBC and lymphoma cells *in vitro*, and suppressed growth of the lymphoma as well as the breast cancer cell-derived xenografts in SCID mice [52, 53]. Another recent report demonstrated that CARP-1/CCAR1 directly binds with LIM-domain protein Zyxin and promotes UV-induced apoptosis in mouse embryonic fibroblasts (MEFs) [56]. Zyxin regulates actin assembly and shuttles between focal adhesions and cell nuclei in respose to mechanical stress. Further, in response to UV-C irradiation, CARP-1/CCAR1 binds to LIM domain of zyxin and promotes its pro-apoptotic activity while expression of zyxin mutant lacking LIM domain failed to bind with CARP-1/CCAR1 and interfered with apoptosis [56]. Consistent with the property of CARP-1/CCAR1 to regulate apoptosis signaling, a couple of studies revealed that CARP-1/CCAR1 is downregulated in a gene expression profiles of tumors derived from rasv12/E1A-transformed MEFs [57], and in peripheral blood cells from neurodegenerative disease, Friedreich's Ataxia patients [58]. Overall, it is increasingly becoming evident that CARP-1/CCAR1 also functions as a tumor suppressor and/or mediator of apoptosis in diverse cancers [3–5, 10, 45, 52–54]. The pathways and interactions involved in pleiotropic signal transduction by CARP-1/CCAR1 that are known to date are summarized in Table 1.

**Table 1:** List of CARP-1/CCAR1 Functions and Interacting Proteins

CARP-1/CCAR-1 functions and interacting partners	References
Transducer of apoptosis signaling	Rishi et al., 2003 [3]
Negative regulator of EGFR signaling	Rishi et al., 2006 [4] Yoo et al., 2004 [43]
Binds and co-activates p53	Kim et al., 2008 [10]
Regulator of steroid/thyroid receptor signaling	Kim et al., 2008 [10]
Positively regulates adipocyte differentiation	Ou et al., 2014 [11]
Binding partner of β-catenin	Ou et al., 2009 [14]
Associated with DBC-1 in NRs transcription	Yu et al., 2011 [25]
Binds to Ngn3 in nucleus	Lu et al., 2012 [27]
Negatively regulated by Necdin	François et al., 2012 [36]
Binding partner of AKAP350A	Kolobova et al., 2009 [39]
Regulates Notch signaling in association with Par-4/THAP1	Lu et al., 2013 [54]
Binding partner of LIM protein Zyxin	Hervy et al., 2010 [56]
Interacts with SAPK p38	Papin et al., 2005 [59]
Interacts with NEMO/IKKγ	Bouwmeester et al., 2004 [60]
Binds and co-activates APC-2 protein	Puliyappadamba et al., 2011 [61]
Interacts with CDC20 and Cdh1	Puliyappadamba et al., 2011 [61]
Involved in apoptosis signaling in Mesothelioma, Medulloblastoma, and Neuroblastoma cells	Ashour et al., 2013 [68] Jamal et al., 2014 [69] Muthu et al., 2014 [70]
Binding partner of DEDD2, FADD, RIPK1 and p43/41 fragment of cleaved Caspase 8	Muthu et al., 2015 [71]

### CARP-1/CCAR1 signaling as a basis for development of novel approaches for tumor suppression

High throughput proteomic studies revealed that CARP-1/CCAR1 interacts with SAPK p38 [59] and NF-κB upstream kinase subunit NEMO/IKKγ [60]. Considering the fact that TAT-mediated transduction of various non-overlapping peptides of CARP-1/CCAR1 suppressed the growth of HBC and lymphoma cells *in vitro* and *in vivo* [52, 53], and CARP-1/CCAR1 co-activates p53 to transduce ADR-dependent apoptosis in breast cancer cells [10], we speculated that CARP-1/CCAR1 likely regulates cell growth and apoptosis signaling by associating with additional key components of cell growth and cell cycle signal transduction pathways. To test this possibility, we performed a yeast two-hybrid screening assay to identify additional binding partners of CARP-1/CCAR1. We found that CARP-1/CCAR1 specifically interacts with Anaphase Promoting Complex/Cyclosome (APC/C) subunit APC-2 protein [61]. Co-immunoprecipitation (co-IP)-western blot (WB) experiments revealed that CARP-1/CCAR1 (896–978) peptide harbored the epitope which interacts with APC-2. Additional co-IP-WB experiments revealed that CARP-1/CCAR1 also interacts with Cdc20 and Cdh1, co-activators of APC/C. The CARP-1/CCAR1 epitope that interacts with Cdc20 or Cdh1 is distinct from its APC-2-interacting epitope [61]. These findings indicate a broader CARP-1/CCAR1 association with APC/C proteome [61]. In light of the fact that APC/C plays a distinct role in various cell cycle checkpoints [62, 63] and deregulation of APC/C and its regulators and substrates has been implicated in tumor progression [64], the components of the APC/C proteome including its co-activator CARP-1/CCAR1 therefore represent attractive targets for design of cell cycle inhibitory strategies with potential for therapeutic use [65–67].

### CARP-1/CCAR1 Functional Mimetics (CFMs)

**Figure 2:**
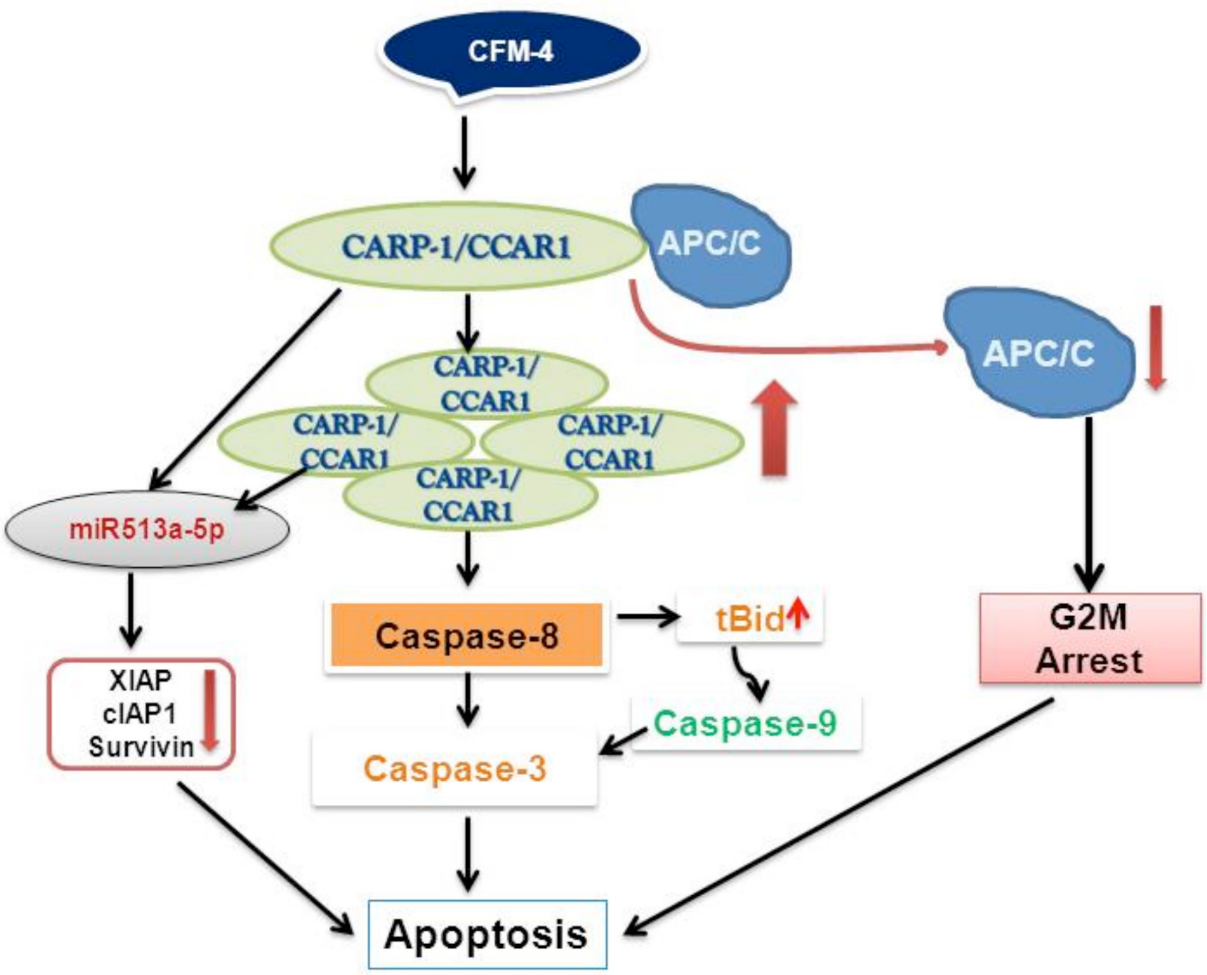
A Schematic of CFM-4-CARP-1/CCAR1-APC/C Signaling

Based on the knowledge of CARP-1/CCAR1 pro-apoptotic signaling in diverse cancer cells, and the fact that CARP-1/CCAR1 binds with APC-2 and co-activates APC/C, while APC/C is a crucial regulator of cell cycle, led us to speculate that disruption of APC/C co-activation by CARP-1/CCAR1 could impact cell cycle progression and ultimately the growth and survival of the cells. To test this possibility, we conducted a high-throughput chemical biology experiment to identify small molecule inhibitors (SMIs) of CARP-1/CCAR1-APC-2 interaction. This experiment yielded multiple, novel SMIs of CARP-1/CCAR1-APC-2 binding, termed CARP-1/CCAR1 Functional Mimetics (CFMs) [61]. CFM-1, CFM-4, and CFM-5 compounds inhibited CARP-1/CCAR1-APC-2 interaction with IC50 values of 4, 1, and 0.75 μM, respectively, while CFM-4 and CFM-5 compounds also bind with CARP-1/CCAR1. Although all the three compounds inhibited growth of a variety of cancer cells including HBC cells *in vitro*, CFM-4 and CFM-5 compounds induced G_2_M cell cycle arrest, promoted loss of cyclin B1, p21^WAF1/CIP1^, p27^KIP1^. CFMs cause increase in CARP-1/CCAR1 levels, and apoptosis in part by activation of caspases-9, -8, and -3 [61]. Moreover, pharmacologic blockage of caspase-8, but not caspase-9, prevented apoptosis by CFM-4 indicating that caspase-8 activation is necessary for apoptosis signaling by CFM-4 [61; Figure 2]. Importantly, knock-down of CARP-1/CCAR1 or APC/C co-activator Cdh1 interferes with apoptosis by CFM-4, suggesting that CARP-1/CCAR1 and Cdh1 play a crucial role in regulating apoptosis by CFMs [61]. Moreover, CFM-4 attenuates proliferation of the ADR or Tamoxifen (TAM)-resistant MCF7 HBC cells without impacting the growth of immortalized, non-tumorigenic human MCF-10A cells [61]. Thus, our discoveries of CARP-1/CCAR1 and CFMs provide a proof-of-concept that CARP-1/CCAR1 signaling could be exploited to identify agents to inhibit breast cancer cells and their drug-resistant variants.

Given the fact that CFMs inhibited growth of breast cancer cells, we further investigated their potential in inhibiting other cancers such as malignant pleural mesothelioma (MPM), medulloblastoma (MB), neuroblastoma (NB) and non-small cell lung cancer (NSCLC) that often have poor prognosis and are known to develop resistance to therapeutic modalities in clinic. Consistent with our previous observations with HBC cells, CFM-4 and CFM-5 compounds inhibited the growth of MPM, MB, NB, and NSCLC cells [68–71] in part by activating apoptosis signaling and diminishing the levels of key cell cycle regulatory proteins including cyclin-B1, c- and N-myc. Both CFM-4 and CFM-5 compounds enhanced expression of CARP-1/CCAR1 along with activation of pro-apoptotic SAPKs (p38α/β and JNK1/2) [68–71]. CFMs, particularly CFM-4, also activated NF-κB signaling by depleting its inhibitors ABIN1 and inhibitory κB (IκB) α and β proteins [68–70], while increasing the expression of pro-apoptotic death receptor (DR) 4 protein. CFM-4 also enhanced expression of serine-phosphorylated cell surface sialo-glycoprotein podoplanin that regulates cell motility and integrity, while promoting cleavage of vimentin in MPM cells [69]. In light of the fact that serine phosphorylation in the short cytoplasmic domain of podoplanin interferes with the processes of cellular motility [72], podoplanin phosphorylation and cleavage of vimentin in the CFM-4-treated MPM cells would suggest for motility and metastasis-inhibitory properties of CFMs [69]. Our gene-array based analysis in MB cells revealed that CFM-4 enhanced the expression of neurotrophin (NTF3), while depletion of NTF3 expression inhibited MB cell apoptosis by CFM-4, suggesting a potential therapeutic window with CFM-4 mediated apoptosis through NTF3 signaling in MB cells [68].

Interestingly, a high-throughput miRNA profiling of malignant pediatric tumor NB cells revealed up-regulation of XIAP-targeting miR513a-5p in response to CFM-4 treatment [70]. Both CFM-4 and CFM-5 compounds significantly abrogated the expression of cell survival associated XIAP1, cIAP1 and survivin proteins in NB as well as in HBC cells. Moreover, expression of anti-miR513a-5p or miR513a-5p mimic interfered with or enhanced, respectively, the apoptosis by CFM-4 in HBC cells [70], suggesting involvement of miR-dependent post-transcriptional targeting of XIAP family of proteins in inhibiting both NB and HBC cell growth in the presence of CFM-4 [70; Figure 2]. Although CFM compounds target podoplanin and vimentin signaling, further *in vitro* studies indicate that CFMs impact ability of the MB, MPM, NB, NSCLC, and HBC cells to grow in soft agar, migrate to close a wound, and invade through the matrigel-coated membranes [68–71].

In an attempt to develop CFMs as potential anti-cancer agent, we performed structure activity relationship (SAR) studies and identified six additional compounds, termed as CFM-4.1 – CFM-4.6, that were structurally similar to our lead compound CFM-4. Among all, CFM-4.6 was very effective in inhibiting the growth of NSCLC (A549, H1299) and MDA-MB-231 triple negative breast cancer (TNBC) cells *in vitro*. Consistent with our earlier studies, both CFM-4 and CFM-4.6 enhanced the CARP-1/CCAR1 expression and activated the pro-apoptotic SAPKs p38α/β and JNK1/2. Additionally, we found that these compounds stimulated the expression of death effector domain containing protein DEDD2. Co-IP-WB studies revealed that CARP-1/CCAR1 directly interacts with DEDD2, FADD, RIPK1 and p43/41 fragment of cleaved caspase 8 [71]. Since activation of caspase 8 is required for apoptosis by CFM-4 [61], and the fact that activation of caspase 8 is often regulated by TNFα receptor (TNFR)/DR family of proteins [73–75], it is likely that CFMs promote formation of an apical, pro-apoptotic sub-complex of CARP-1/CCAR1-DEDD2-FADD-RIPK1-p43/41 caspase 8 proteins. Moreover, since CARP-1/CCAR1 and DEDD2 are known to shuttle between cytosol and nucleus [3, 76, 77], it is possible that CFMs induce apoptosis by stimulating levels of CARP-1/CCAR1 and DEDD2 proteins and their intracellular shuttling to transduce apoptosis [71]. CFM-4, CFM-5, and CFM-4 analog CFM-4.6 disrupt formation of tubules by human vascular endothelial cells (HUVECs) *in vitro*, suggesting that these compounds likely also possess anti-angiogenic properties [71].

### CFM-4 nano lipid formulations (CFM-4 NLFs)

To demonstrate the therapeutic potential of CFMs, preclinical studies in xenograft models of MB, MPM, and breast cancers were carried out. Direct, intravenous administration of CFM-4 cocktail failed to inhibit growth of subcutaneously xenografted tumor cells in immunocompromised mice [71]. Since, nanolipid based formulations often increase the physicochemical stability of both the drug molecules and particulate system, and have higher drug-loading capacity, we next investigated whether a nanolipid based formulation of CFM-4 could be generated and utilized to further test the therapeutic potential of CFM-4 compound. A natural, biocompatible cationic polysaccharide Chitosan-based nanolipid formulation of CFM-4 (CFM-4 NLF) was prepared as described before [71, 78]. CFM-4 NLF had superior bioavailability and pharmacokinetics when compared with the CFM-4 free drug [71], and inhibited growth of xenografted TNBC and NSCLC tumors in nude mice, when administered orally [71]. Biochemical analyses of representative NSCLC and TNBC tumor xenografts from placebo (control) or CFM-4 NLF-treated animals revealed accumulation of CFM-4 NLF in tumors of treated animals. Additionally, Immuno-histological analyses showed elevated levels of CARP-1/CCAR1 and fragmented DNA in tumors of CFM-4 NLF-treated animals when compared with those of placebo-treated controls [71]. These findings provide a proof-of-concept that CFMs possess therapeutic potential, and function *in vivo* in part by stimulating CARP-1/CCAR1 levels and apoptosis.

## CONCLUSION AND FUTURE PERSPECTIVE

Overall, it is becoming increasingly evident that CARP-1/CCAR1 regulates signaling ranging from co-activation of physiological responses to steroids, processes of cellular differentiation and homeostasis in different tissues, to the chemotherapy-dependent apoptosis signaling with or without co-activation of tumor suppressor p53. Identification of CFMs through the chemical biology strategies provides a further proof-of-concept that CARP-1/CCAR1 and an aspect of its signaling could be exploited to inhibit cancer cells. The lead compound CFM-4 inhibits growth of a variety of cancer cells including the chemo-resistant breast cancer cells without impacting growth of immortalized, non-tumorigenic breast epithelial cells. Although CFM-4 and CFM-5 compounds function in part by binding and elevating cellular levels of CARP-1/CCAR1, apoptosis by radiation, chemotherapy (such as ADR, Etoposide, or Gefitinib), the CFM-4 and CFM-5 compounds, and the physiological responses of various steroids nevertheless require CARP-1/CCAR1. Thus targeting of CARP-1/CCAR1 could allow for physiological fine tuning of steroid responses, the discovery of CFMs and their future analogs on the other hand offer novel tools for design of anti-cancer strategies for combating a variety of cancers including the TNBCs and their drug-resistant variants. The promising *in vitro* and *in vivo* pre-clinical studies thus far provide a compelling rationale for further exploration of the pleiotropic cell growth and apoptosis signaling by CARP-1/CCAR1, and potential of CFMs as novel agents for optimization of anti-cancer therapeutic strategies. Since current treatment options for TNBCs as well as drug-resistant cancers of breast, lung, MPM, and neurological origins are limited, discovery and development of molecules based on CARP-1/CCAR1 signaling could yield novel tools for management and treatment of cancers and thus potentially serve an urgent and unmet need.
